# BOLD Imaging in Awake Wild-Type and Mu-Opioid Receptor Knock-Out Mice Reveals On-Target Activation Maps in Response to Oxycodone

**DOI:** 10.3389/fnins.2016.00471

**Published:** 2016-11-03

**Authors:** Kelsey Moore, Dan Madularu, Sade Iriah, Jason R. Yee, Praveen Kulkarni, Emmanuel Darcq, Brigitte L. Kieffer, Craig F. Ferris

**Affiliations:** ^1^Department of Psychology, Center for Translational NeuroImaging, Northeastern UniversityBoston, MA, USA; ^2^Brain Imaging Center, Douglas Hospital Research Institute, McGill UniversityMontreal, QC, Canada

**Keywords:** oxycodone, opioid receptors, mu-opioid receptor, knockout mouse, addiction, target activation map, BOLD imaging, BOLD fMRI

## Abstract

Blood oxygen level dependent (BOLD) imaging in awake mice was used to identify differences in brain activity between wild-type, and Mu (μ) opioid receptor knock-outs (MuKO) in response to oxycodone (OXY). Using a segmented, annotated MRI mouse atlas and computational analysis, patterns of integrated positive and negative BOLD activity were identified across 122 brain areas. The pattern of positive BOLD showed enhanced activation across the brain in WT mice within 15 min of intraperitoneal administration of 2.5 mg of OXY. BOLD activation was detected in 72 regions out of 122, and was most prominent in areas of high μ opioid receptor density (thalamus, ventral tegmental area, substantia nigra, caudate putamen, basal amygdala, and hypothalamus), and focus on pain circuits indicated strong activation in major pain processing centers (central amygdala, solitary tract, parabrachial area, insular cortex, gigantocellularis area, ventral thalamus primary sensory cortex, and prelimbic cortex). Importantly, the OXY-induced positive BOLD was eliminated in MuKO mice in most regions, with few exceptions (some cerebellar nuclei, CA3 of the hippocampus, medial amygdala, and preoptic areas). This result indicates that most effects of OXY on positive BOLD are mediated by the μ opioid receptor (on-target effects). OXY also caused an increase in negative BOLD in WT mice in few regions (16 out of 122) and, unlike the positive BOLD response the negative BOLD was only partially eliminated in the MuKO mice (cerebellum), and in some case intensified (hippocampus). Negative BOLD analysis therefore shows activation and deactivation events in the absence of the μ receptor for some areas where receptor expression is normally extremely low or absent (off-target effects). Together, our approach permits establishing opioid-induced BOLD activation maps in awake mice. In addition, comparison of WT and MuKO mutant mice reveals both on-target and off-target activation events, and set an OXY brain signature that should, in the future, be compared to other μ opioid agonists.

## Introduction

Oxycodone (OXY) is a powerful analgesic initially prescribed for the management of acute pain, pain following surgery, and pain associated with palliative care. Laws allowing the use of opioid analgesics in the management of non-cancerous, chronic pain led to the escalation of OXY use, and not unexpectedly its abuse (Manchikanti et al., 2010). Indeed, there is accumulating evidence that OXY is more addictive than morphine (Stoops et al., 2010; Comer et al., 2013). Opioid addiction in the United States accounted for nearly 19,000 overdose deaths in 2014 with OXY responsible for a majority of these fatalities (Center for Disease Control and Prevention, 2015). The cost to the health care system is staggering and was estimated be $55.7 billion annually in 2007 and has only risen since (Birnbaum et al., 2011).

The three opioid receptors Mu (μ), kappa (κ), and delta (δ), are located throughout the brain across all mammalian species thus studied (Dhawan et al., 1996). Opioid signaling in the brain involves not only the regulation of pain, but the modulation of behavior associated with reward, depression, anxiety, and obviously addiction (for reviews, see Al-Hasani and Bruchas, 2011; Pradhan et al., 2012; Lutz and Kieffer, 2013a,b). Upon interaction with opioid receptors, preferably the μ receptor (Yoburn et al., 1995), OXY has been shown to affect dopamine, as well as GABA transmission (Vander Weele et al., 2014; Takasu et al., 2015). The analgesic effects of morphine are mediated through μ receptors as shown in human and animal studies using specific μ-receptor antagonists in the presence of a painful stimulus and in studies on Mu-opioid knock-out (MuKO) mice (Matthes et al., 1996; Sora et al., 1997). Indeed, MuKO mice showed lack of morphine analgesia, rewarding effect and physical dependence, indicating that a single receptor mediates all biological effects of the prototypic opiate (Matthes et al., 1996). Whether this is also true for OXY has not been tested in MuKO mice, as yet. Ross and Smith reported a κ-mediated analgesic effect of OXY given intracerebroventricularly to rats that could only be blocked with a selective κ receptor antagonist but not μ or δ antagonists (Ross and Smith, 1997). Finally, μ-opioid analgesics (but not endogenous μ-opioid peptides), such as morphine, fentanyl, and OXY promote inhibition of thalamic neuronal activity; the effects were not affected by μ-opioid receptor antagonist treatment, nor the deletion of the μ-opioid receptor gene (Hashimoto et al., 2009), indicating possible off-target effects for these μ-opioid agonists.

The present study using the MuKO mouse (Matthes et al., 1996) provided an opportunity to evaluate the global effect of OXY on brain activity in a WT mouse and the contribution of the μ receptor to this pattern of activity. With non-invasive ultra-high field, functional magnetic resonance imaging (fMRI) in awake animals it is possible to image changes in brain activity across distributed, integrated neural circuits with high temporal, and spatial resolution (Ferris et al., 2011). When combined with the use of 3D segmented, annotated, brain atlases, and computational analysis it is possible to reconstruct the activity of OXY on the neural circuits of pain, reward, and addiction that demand our understanding. As such, MuKO and WT mice were scanned awake and BOLD activation in response to OXY was assessed.

## Methods

### Animal care

Male *Oprm1*^+/+^ and *Oprm1*^−/−^ mice (Matthes et al., 1996) were bred in-house at the Douglas Hospital Research Center, Montreal, Canada. At ages 10–12 weeks, animals were transferred to Northeastern University, Center for Translational NeuroImaging. Mice were left undisturbed for 1 week before the start of an experimentation, and were allowed access to food and water *ad libitum* while housed in groups of up to four per cage with mice of the same genotype. Animals were cared for in accordance with the guidelines published in the Guide for the Care and Use of Laboratory Animals (National Institutes of Health Publications No. 85–23, Revised 1985) and adhered to the National Institutes of Health and the American Association for Laboratory Animal Science guidelines. The protocols used in this study were in compliance with the regulations of the Institutional Animal Care and Use Committee at Northeastern University.

### Awake mouse imaging system

**Figure 1:**
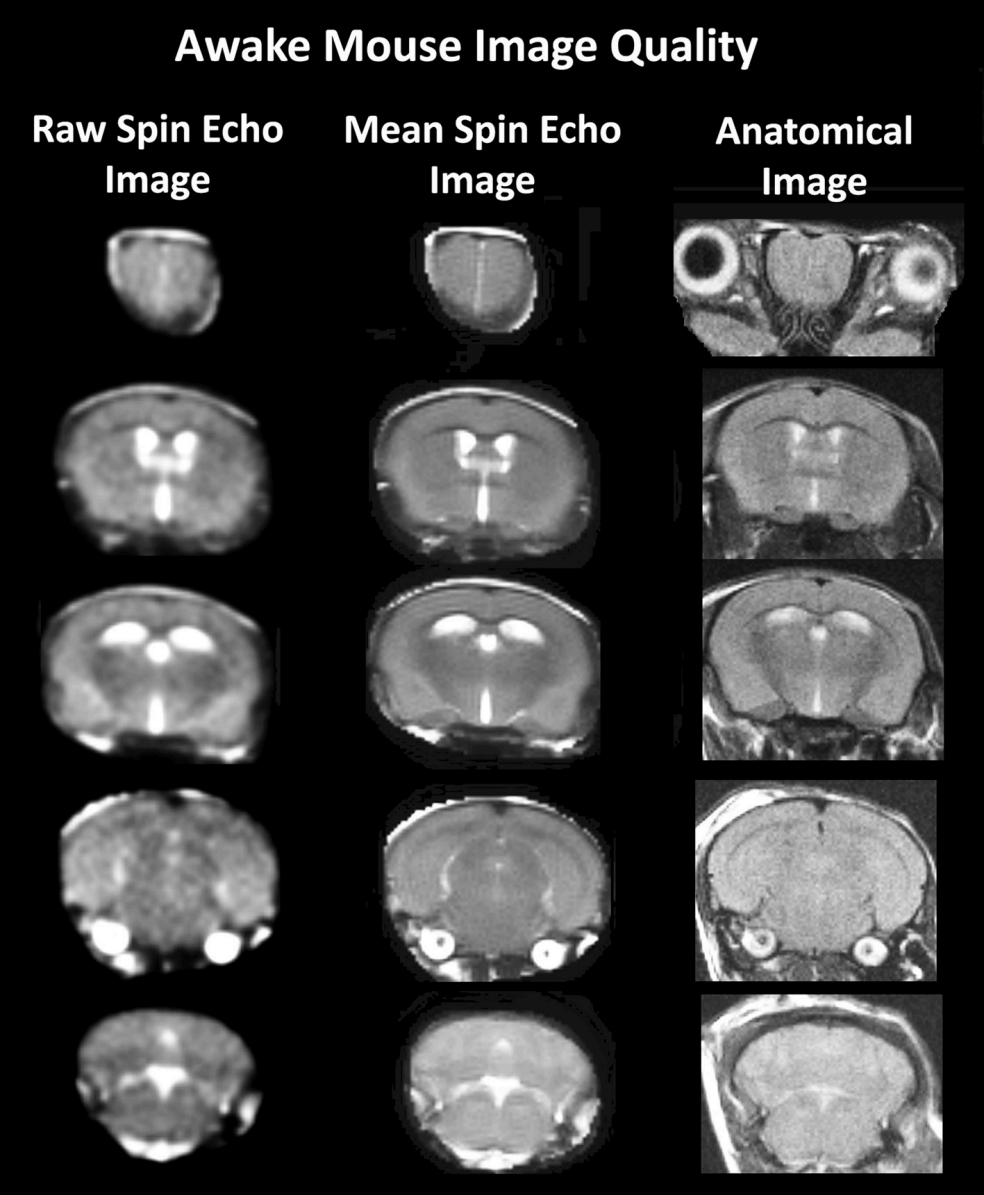
**Anatomical fidelity**. Shown are representative examples of brain images collected during a single imaging session using a multi-slice spin echo, RARE (rapid acquisition with relaxation enhancement) pulse sequence. The column on the right shows axial sections collected during the anatomical scan taken at the beginning of each imaging session using a data matrix of 256 × 256, 20 slices in a field of view of 2.5 cm. The column on the left shows the same images but collected for functional analysis using HASTE, a RARE pulse sequence modified for faster acquisition time. These images were acquired using the same field of view and slice anatomy but a larger data matrix of 96 × 96. The images in the middle column have been smoothed during pre-processing. Note, the anatomical fidelity between the functional images and their original anatomical image. The absence of any distortion is necessary when registering the data to atlas to resolve 122 segmented brain areas.

A detailed description of the awake mouse imaging system has been published previously (Ferris et al., 2014). The quadrature transmit/receive volume coil (ID 38 mm) provides excellent anatomical resolution and signal-noise-ratio for voxel-based blood oxygen level dependent (BOLD) fMRI (see Figure 1). The unique design of the holder essentially stabilizes the head in a cushion, minimizing any discomfort normally caused by ear bars, and pressure points used to immobilize the head for awake animal imaging.

### Acclimation

A week prior to the first imaging session, all animals were acclimated to the imaging protocol, i.e., head restraint and noise of the scanner before the imaging session. Mice were secured in the holding system while anesthetized with 2–3% isoflurane. Following cessation of isoflurane, fully conscious mice were put into a “mock scanner” (a black box with a tape recording of MRI pulses) for 30 min for 4 consecutive days. Acclimation in awake animal imaging significantly reduces physiological effects of the autonomic nervous system including heart rate, respiration, corticosteroid levels, and motor movements (King et al., 2005) helping to improve contrast-to-noise and image quality.

### Oxycodone preparation and administration

Oxycodone (OXY) was purchased from Sigma Chemical (St. Louis MO, USA) and dissolved in 0.9% NaCl for intraperitoneal (IP) injections. To deliver drug remotely during the imaging session, a poly-ethylene tube (PE-20), ~30 cm in length, was positioned in the peritoneal cavity. The dose of 2.5 mg/kg of OXY was taken from the study of Zhang and colleagues that reported a significant increase in dopamine in the striatum of the mouse within 20 min of IP drug injection (Zhang Y. et al., 2009).

### Imaging acquisition and pulse sequence

Experiments were conducted using a Bruker Biospec 7.0T/20-cm USR horizontal magnet (Bruker, Ettlingen, Germany) and a 20-G/cm magnetic field gradient insert (ID = 12 cm) capable of a 120-μs rise time. At the beginning of each imaging session, a high-resolution anatomical data set was collected using the RARE pulse sequence (20 slice; 0.75 mm; FOV 2.5 cm; data matrix 256 × 256; repetition time (TR) 2.1 s; echo time (TE) 12.4 ms; Effective TE 48 ms, NEX 6; 6.5 min acquisition time). Functional images were acquired using a multi-slice HASTE pulse sequence (Half Fourier Acquisition Single Shot Turbo Spin Echo). With this sequence it is possible to collect twenty, 0.75 mm thick, axial slices in <6 s. With a FOV of 2.5 cm and a data matrix of 96 × 96, the in-plane pixel functional resolution for these studies was 260 μm^2^.

For the functional scans, maintaining neuroanatomical fidelity was a priority since: (1) we are imaging activity in brains very small in size, and (2) awake mice (even when acclimated) exhibit some degree of motion. The neuroanatomical fidelity of our functional scans can be seen when presented alongside the same animal's anatomical scan (see example, Figure 1). To achieve anatomical fidelity across scans that encompass the whole brain, we chose to use spin echo pulse sequences that were developed for awake imaging in rats and mice (Ferris et al., 2011). In relation to the more common gradient echo BOLD, spin echo BOLD offers several major advantages: (i) the potential of improved functional spatial resolution since functional signal changes are localized to the capillary bed, and (ii) elimination of magnetic susceptibility artifacts, particularly signal dropout at the interface of air-filled sinus and gray matter (Norris, 2006). Specifically, we used a single-shot fast spin echo/rapid acquisition with relaxation enhancement (FSE/RAREst) pulse sequence with half-Fourier transform that provided reasonable in-plane spatial resolution (260 μm2), sufficiently thin slices (700 μm/slice), and scans of the entire brain (20 slices) in 6 s. The major disadvantage to spin echo BOLD is low sensitivity, but this is addressed by using a high magnetic field strength (7T). In addition, and importantly, FSE/RAREst scans run at high magnetic field strengths (7T and above) result in BOLD signal dominated by the extravascular dynamic averaging component of the T2-weighted signal (Ugurbil et al., 2000; Duong et al., 2003; Yacoub et al., 2003; Zhang N. et al., 2009). The extravascular signal surrounding capillary beds and small vessels is more reflective of the metabolic changes in brain parenchyma than signal from large draining veins and thus provides a more accurate measure of neuronal activity (Yacoub et al., 2007). A second disadvantage of spin echo BOLD is that, even with the advancement of parallel imaging techniques combined with partial Fourier acquisition and the associated shortening of scan times, complete coverage of a large brain, like the human is not possible at sufficiently short repetition times (TR) for event-related fMRI. However, we are able to circumvent this disadvantage given the substantially smaller brain size of rodents and the use of a single epoch stimulus-presentation period.

Multi-slice FSE/RAREst using a partial Fourier acquisition with a 9/16 ratio pulse sequences were run with Bruker Paravision v.5.1. With this pulse sequence we imaged the entire brain, collecting 22 axial slices per repetition, at 1.0 mm thick, in 6 s repetition intervals [22 slices; slice thickness, 1.0 mm; FOV 3.0 cm; data matrix 96 × 96; repetition time (TR) 6.0 s; echo time (TE) 3.0 ms; effective TE 45 ms; RARE factor, 62; NEX, 1]. With a FOV of 3.0 cm and a data matrix of 96 × 96, the in-plane pixel functional resolution for these studies was 312 μm^2^. In automated fashion, Paravision v.5.1 finds the basic frequency, shims, determines power requirements for 90° and 180° pulses, and sets the receiver gain proportionally. Each single-epoch, event-related scanning session was run in continuous fashion.

### Data analysis

Data are coregistered to a mean functional image using SPM8's coregistrational code with the following parameters: Quality: 0.97, Smoothing: 0.35 mm, Separation: 0.5 mm. Gaussian smoothing was performed with a FWHM of 0.8 mm. Images were aligned and registered to a 3D mouse brain atlas, which is segmented and labeled with 122 discrete anatomical regions (Ekam Solutions, Boston MA). The alignment process was facilitated by an interactive graphic user interface. The registration process involved translation, rotation, and scaling independently and in all three dimensions. Matrices that transformed each subject's anatomy were used to embed each slice within the atlas. All pixel locations of anatomy that were transformed were tagged with major and minor regions in the atlas. This combination created a fully segmented representation of each subject within the atlas. The composite statistics were built using the inverse transformation matrices. Each composite pixel location (i.e., row, column, and slice), pre-multiplied by [*Ti*]^−1^, mapped it within a voxel of subject (*i*). A tri-linear interpolation of the subject's voxel values (percentage change) determined the statistical contribution of subject (*i*) to the composite (row, column, and slice) location. The use of [*Ti*]^−1^ ensured that the full volume set of the composite was populated with subject contributions. The average value from all subjects within the group determined the composite value.

Using voxel-based analysis, the percent change in BOLD signal for each independent voxel was averaged for all subjects. Each scanning session consisted of data acquisitions (whole brain scans) with a period of 6 s (TR) each for a total lapse time of 20 min. The control window was the first 50 scan repetitions (5 min baseline) while the treatment stimulation window was 50–200 (min 5–20). Statistical *t*-tests were performed on each voxel (ca. 15,000 in number) of each subject within their original coordinate system with a baseline threshold of 2% BOLD change to account for normal fluctuation of BOLD signal in the awake rodent brain (Brevard et al., 2003). As a result of the multiple *t*-test analyses performed, a false-positive detection controlling mechanism was introduced (Genovese et al., 2002). This subsequent filter guaranteed that, on average, the false-positive detection rate was below our cutoff of 0.05. The *t*-test statistics used a 95% confidence level, twotailed distributions, and heteroscedastic variance assumptions.

A composite image of the whole brain representing the average of all subjects was constructed for each group for analyses of 122 brain areas, allowing us to look at each brain area separately to determine the BOLD change and the number of activated voxels in each area. Volume of activation was compared across experimental groups using the non-parametric Kruskall-Wallis test statistic. Brain areas were considered statistically different between experimental groups when comparison produced *P*-values less than or equal to our cutoff of 0.05. *Post-hoc* analyses were performed with a Wilcoxon rank-sum test.

Group difference for the change in BOLD signal over time (time course analysis) were assessed by one-way ANOVA at each time point to determine when groups started to diverge from one another following the injection. Tukey-Kramer *post-hoc* tests were run in the event of the first significant result to determine the onset of pairwise group differences. Timing of change from baseline in % BOLD was assessed by group using sequential paired *t*-tests with a hypothesized difference of 0.

### Normalization of volume of activation

The differences BOLD signal change in *Oprm1* +/+ wild-type (WT) and *Oprm1* −/− μ-opioid knock-out (MuKO) mice are reported in terms of volume of activation or number of voxels per brain area to control for any differences in brain volume that may occur between genotypes. In this study, on average, the brain size in MuKO mice was slightly less than WT but not significantly different (see Figure 1S) although there were a few brain areas e.g., ventral tegmental area and glomerular layer of the olfactory bulb that did show a significant difference across genotypes. By normalizing to volume of activation we can compare across different genotypes or within groups among different regions. Normalized volume of activation was computed using following formula.

(1) $$\begin{array}{l} \text{Normalized number of voxels in ROI}\\ \;=\frac{\text{Number of activated voxels in ROI}\times \text{ 100}}{\text{Total number of Voxels in ROI}} \end{array}$$

### Calculating the volumes of different brain areas

The volume of each brain area was determined from the high resolution anatomical scan taken at the beginning of each scanning session for each subject. The 3D segmented atlas provides the precise number of voxels (3D pixels) that combine to fill the volume of each of the 122 brain regions. The dimensions of each voxel are calculated from the slice thickness (0.75 mm), Voxel width (FOV in X direction/Number of voxels in X direction) and Voxel height (FOV in Y direction/Number of voxels in Y direction) using the formula:

(2) $$\begin{array}{l} Volume\;of\;Voxel\;=\;voxel\;width\;\times \;voxel\;height\\ \;\times \;slice\;thickness \end{array}$$

[ca. 0.097 × 0.097 × 0.750 mm = 0.00706 mm^3^]. Total number of voxels in each brain area was multiplied by volume of voxel to compute total volume of brain region.

### Carbon dioxide challenge

**Figure 2:**
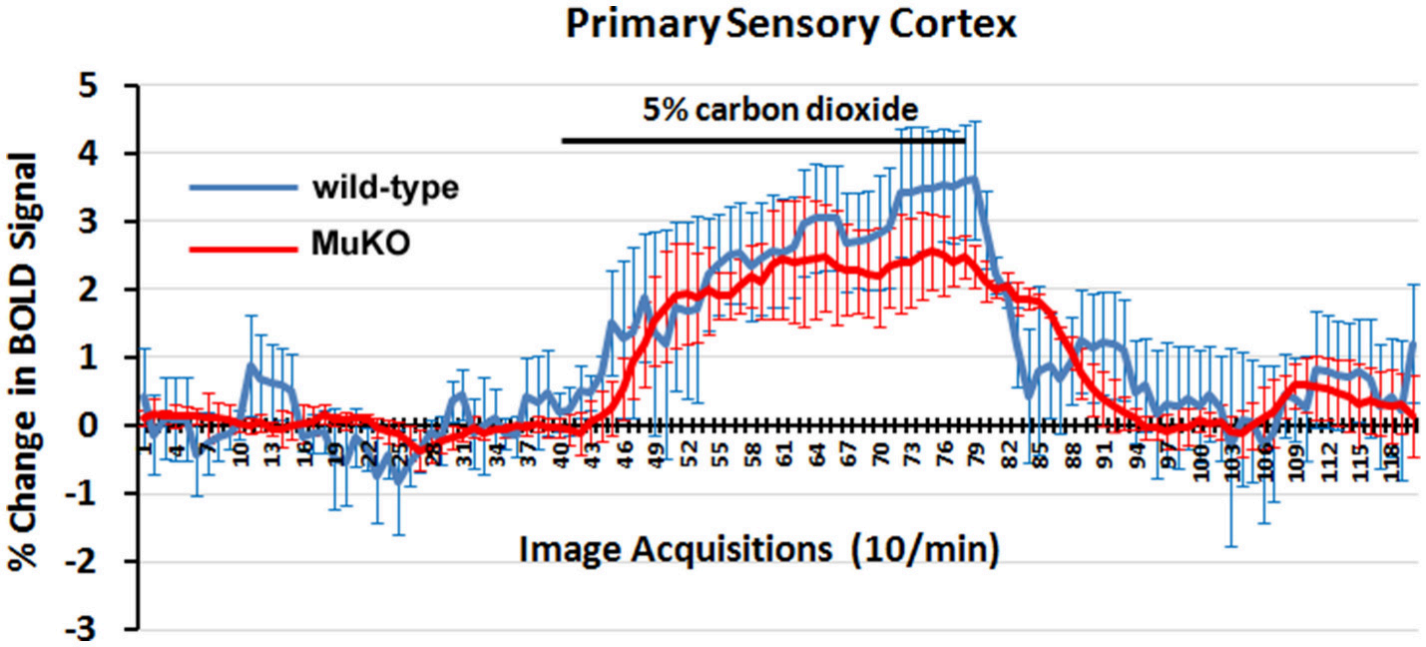
**BOLD signal change to carbon dioxide challenge**. Shown are time-course data for each WT and MuKO mice for the percentage change in BOLD signal intensity in the somatosensory cortex in response to the challenge of 5% carbon dioxide. Each image acquisition is the mean ± SEM.

To assess the strength of the BOLD signal in mice using HASTE and to further characterize any differences between WT (*n* = 5) and MuKO (*n* = 5) mice in terms of cerebrovascular reactivity animals were challenged with a 5% CO_2_ as a stimulus for a surrogate BOLD response (see Figure 2). Carbon dioxide causes a direct relaxation of cerebrovascular smooth muscle, causing a passive dilation with a subsequent increase in cerebral blood flow. To this end, mice were imaged for a total of 5 min with presentation of 5% CO_2_ in ambient air at 2.5 min into the scanning session. The presentation of CO_2_ is facilitated by the design of the head holder. The front incisors of the mouse are locked onto a bite bar by pulling the snout into a beveled nose cone. The cone is perforated so as not to restrict the flow of air from the nostrils or mouth. A hollow tube extends from the tip of the nose cone providing a route for administering carbon dioxide gas. Data were analyzed using a repeated measures ANOVA followed by Fisher's protected least significant difference to limit experiment-wise error when performing pairwise comparisons between genotypes. There were no significant differences between genotypes.

## Results

Shown in Table 1 are the positive BOLD signal changes represented as a percentage of the total brain area volume (i.e., number of voxels activated, divided by the total number of voxels in the 3D volume of interest, times 100) for WT and MuKO mice in response to saline vehicle or 2.5 mg/kg OXY. The brain areas are ranked in order of their significance and are truncated from a larger list of 122 regions of activation (for complete list see Table 1S in Supplementary Material). With a Kruskall-Wallis multiple comparison analysis WT mice treated with oxycodone showed 72 out of 122 brains areas to be significantly different from WT mice treated with saline vehicle. *Post-hoc* analysis showed there were no significant differences between the vehicle group and MuKO mice given OXY. Most of the significance was attributed to WT mice given OXY as compared to the vehicle group. In Table 2 are the negative BOLD signal changes, i.e., number of voxels that showed a significant decline in BOLD signal using the multiple comparison analyzes. This truncated table shows 16 out of 122 brains areas that were significantly different (for complete list see Table 2S in Supplementary Material). Again post hoc analysis showed there were no significant differences between the vehicle group and MuKO mice given OXY.

**Table 1:** **Positive BOLD volume of activation in oxycodone challenge**.

**Oxycodone in WT vs. oxycodone in Mu opioid −/−**				
**Brain Areas**	**Veh Med**	**WT oxy Med**	**KO oxy Med**	***P*****-val**
**POSITIVE BOLD VOLUME OF ACTIVATION**				
9th cerebellar lobule	10	58	10	0.001
Facial nucleus	0	50	21	0.001
Locus ceruleus	0	100	0	0.002
Reuniens thalamic area	0	70	2	0.003
Inferior colliculus	4	52	16	0.003
Central amygdaloid area	0	50	4	0.004
Deep cerebellar nuclear area	0	63	13	0.005
Glomerular layer	21	36	7	0.005
External capsule	0	31	10	0.006
Intermediate reticular area	9	32	20	0.006
3rd cerebellar lobule	0	32	15	0.006
CA3 hippocampus	0	43	9	0.006
Lateral paragigantocellularis	2	44	23	0.007
Extended amydala	0	37	5	0.007
Simple lobule cerebellum	6	33	16	0.007
4th cerebellar lobule	0	24	16	0.008
Principal sensory n. trigeminal	7	42	16	0.008
Olivary complex	0	39	11	0.008
Lateral caudal hypothalamus	0	48	16	0.009
Paramedian lobule cerebellum	5	45	18	0.009
subiculum hippocampus	10	44	7	0.01
Paraventricular hypothalamus	0	42	0	0.01
8th cerebellar lobule	5	52	12	0.01
2nd cerebellar lobule	6	41	12	0.011
Prepositus area	0	42	7	0.011
Solitary tract area	11	40	6	0.011
Parabrachial area	7	53	20	0.012
Rostral piriform ctx	12	29	9	0.012
medial preoptic area	2	37	19	0.012
10th cerebellar lobule	0	37	14	0.012
Orbital ctx	4	42	4	0.013
Dentate gyrus hippocampus	3	38	13	0.015
CA1 hippocampus	4	31	10	0.016
Insular rostral ctx	17	34	15	0.016
Crus of ansiform lobule	19	40	22	0.016
Gigantocelllaris reticular area	3	29	21	0.016
Lateral rostral hypothalamus	2	36	11	0.017
Ventral thalamic area	0	31	18	0.017
Lemniscal area	0	42	19	0.018
Ventral pallidum	0	25	2	0.02
Primary motor ctx	20	39	7	0.021
Secondary motor ctx	17	43	14	0.021
5th cerebellar lobule	1	40	18	0.021
Medial amygdaloid area	1	30	16	0.021
Globus pallidus	0	29	7	0.022
6th cerebellar lobule	30	48	27	0.022
Ventral tegmental area	0	25	2	0.023
Visual 1 ctx	24	44	21	0.023
Dorsal medial hypothalamus	0	38	16	0.024
Primary somatosensory ctx	14	29	13	0.026
Lateral geniculate	0	36	20	0.026
Basal amygdaloid area	11	44	27	0.027
Vestibular area	12	49	29	0.027
Granular cell layer	12	28	3	0.027
7th cerebellar lobule	6	51	16	0.029
Substantia nigra	0	47	13	0.03
Ambiguus area	13	54	18	0.031
Caudate putamen	2	31	5	0.031
Pedunculopontine tegmentum	10	43	26	0.032
Caudal piriform ctx	33	48	30	0.032
Reticulotegmental nucleus	0	22	17	0.033
Anterior amygdaloid area	0	23	4	0.035
Spinal trigeminal n. area	19	35	22	0.038
Prelimbic ctx	7	39	9	0.038
Lateral lemniscus	4	32	19	0.038
Anterior thalamic area	4	47	22	0.039
Parvicellular reticular area	14	40	16	0.041
Lateral septal area	2	38	12	0.041
Superior colliculus	3	31	16	0.042
Posterior hypothalamic area	0	35	0	0.045
Lateral preoptic area	0	56	0	0.046
Fimbria hippocampus	10	43	10	0.046
Medial geniculate	0	46	13	0.046
Pontine reticular nucleus oral	7	26	9	0.05

*Shown is a truncated list of 122 brain areas and their median (med) number of voxels as a percentage of the total Brain Area volume (i.e., number of voxels activated, divided by the total number of voxels in the 3D volume of interest, times 100) for wild-type given saline vehicle (Veh) wildtype given oxycodone (WT Oxy) and μ receptor knock-out mice given oxycodone (KO Oxy). These volumes of activation for each brain region across genotypes were analyzed using a Newman-Keuls multiple comparisons test statistic and ranked in order of their significance. P-values are presented on the far right column*.

**Table 2:** **Negative BOLD volume of activation in oxycodone challenge same as Table 1**.

**Oxycodone in WT vs. oxycodone in Mu opioid −/−**				
**Brain areas**	**Veh Med**	**WT oxy Med**	**KO oxy Med**	***P*****-val**
**NEGATIVE BOLD VOLUME OF ACTIVATION**				
CA1 hippocampus	0	7	6	0.006
Anterior hypothalamic area	0	12	1	0.01
Olfactory tubercles	0	20	0	0.01
Central medial thalamic area	0	0	0	0.012
Reuniens thalamic area	0	0	0	0.012
Ventral medial hypothalamus	0	19	0	0.012
Lateral reticular area	0	15	0	0.022
Medial amygdaloid area	0	9	1	0.023
Cuneate area	0	22	0	0.031
Insular caudal ctx	0	14	4	0.036
Reticular thalamic area	0	8	0	0.041
CA3 hippocampus	0	5	5	0.043
Ventral pallidum	0	11	0	0.043
Accumbens core	0	8	0	0.044
Globus pallidus	0	12	7	0.047
Paramedian lobule	0	13	2	0.049
Anterior amygdaloid area	0	17	4	0.052

### On-target effects of OXY on μ-opioid receptor circuitry

**Figure 3:**
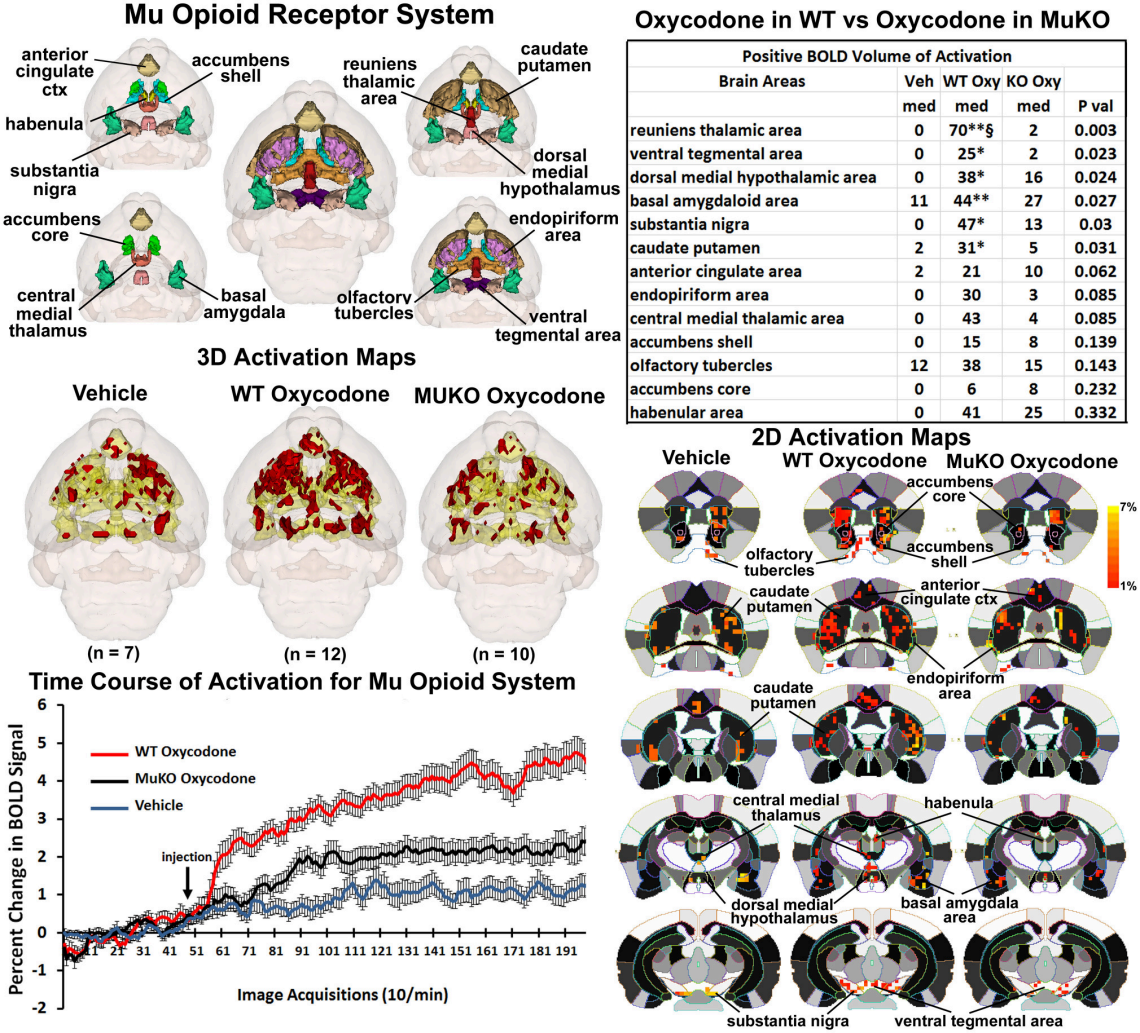
**Mu opioid receptor system**. Shown in the top left are 3D colored volumes of 13 areas in the brain noted to have a high density of μ opioid binding sites. The central image is a coronal view of a translucent shell of the mouse brain showing the total composite and location of the different 3D volumes of interest. Surrounding this are different layers showing a clockwise, caudal (deepest, lower left) to dorsal perspective of the different brain volumes. The color-coded volumes are coalesced into a single volume shown in yellow below for each of the three experimental conditions. The number of animals contributing to the data for each experimental condition are shown in parentheses. Once fully registered and segmented, the statistical responses for each animal are averaged on a voxel-byvoxel bases. Those averaged voxels that are significantly different from baseline for positive BOLD are show in their appropriate spatial location coalesced as a 3D volume. Below on the right are 2D activation maps from the mouse brain atlas showing the precise location of the significantly altered positive (red) voxels following OXY for each experimental condition. These are the same 3D data but shown in a 2D perspective. The vertical color strip shows the scale of the positive BOLD signal change. The table in the upper right lists the 13 areas having a high density of μ opioid binding. The columns show the median (med) number of significant voxels for each brain area for each experimental condition. The voxel numbers for each condition were analyzed using a Newman-Keuls multiple comparisons test statistic followed by *post-hoc* analyses using a Wilcoxon rank-sum test for individual differences. All areas are ranked in order of their significance. There were no significant differences in the volume of activation between vehicle (Veh) and MuKO (KO Oxy) conditions. WT mice given OXY showed significance from Veh (^*^p <0.05, ^**^p <0.01) and from MuKO conditions (§*p* <0.05). Shown in the lower left are time course data for the percent change in positive BOLD signal for each experimental condition. Each acquisition is the mean and SEM of the combined signal from the brains areas that were significantly different as reported in the table.

It was anticipated that many of these activated brain regions would be associated with areas of high μ-opioid receptor density. The distribution of the μ binding sites in the mouse is widespread appearing throughout the neocortex, basal ganglia, thalamus, amygdala, and brain stem (Moskowitz and Goodman, 1984; Slowe et al., 1999; Erbs et al., 2015). A putative neural circuit of those areas showing the highest concentrations of receptor are shown in Figure 3. The images at the top left depict the location of 13 3D brain volumes in the mouse having a high density of μ receptor binding. These areas are color-coded and annotated. These same areas are coalesced into a single volume (yellow) shown in the lower 3D images depicting activation maps following the IP injections of saline vehicle, OXY in WT mice and OXY in MuKO mice. Areas in red are the composite average of the significant increase in volumes of activation (number of voxels in a brain area) for positive BOLD from all mice for each condition. The changes in positive BOLD in the different areas of the μ-opioid receptor neural circuit are better viewed in the 2D activation maps shown the lower right in Figure 3. The precise location of the positive voxels are shown registered to the mouse MRI atlas. These composites are the average number of voxels showing a significant increase above baseline for vehicle (*n* = 7), WT oxycodone (*n* = 12), and MuKO oxycodone (*n* = 10).

The median (Med) number of positive voxels activated for each experimental condition is reported in the table to the upper right. These brain areas are ranked in order of their significance. Of the 13 areas having a high density of OXY binding sites, six were significantly activated by OXY in WT mice as compared saline vehicle. Only the area of the reuniens thalamus was significantly different between WT and MuKO treated with OXY while there were no significant differences between vehicle and MuKO mice. Essentially, the absence of the μ receptor does not eliminate the activation of this neural circuit with OXY treatment, however, the activation is so low that it is not significantly different from vehicle. However, the significant differences between WT OXY and MuKO OXY conditions are clearly delineated in the time course plots depicting the change in positive BOLD signal following vehicle and OXY treatments shown in the lower left of Figure 3. A repeated measures one-way ANOVA showed a significant main effect for group x time, *F*_(2, 398)_ = 3.17, *p* <0.0001. Each experimental condition showed a significant increase from baseline within the first min of IP injection [Paired *t*-test: Baseline (40–50) vs: Vehicle repetition 56 (*p* = 0.041); WT OXY repetition 57 (*p* = 0.005); MuKO OXY repetition 62 (*p* = 0.038)]. WT OXY diverged and remained separate from MuKO OXY and vehicle at repetition 60 (*p* = 0.013). MuKO OXY diverged and remained separate from vehicle at repetition 86 (*p* <0.001). Hence, there is some level of arousal and BOLD signal change that accompanies the I.P injection as shown for vehicle. However, over the 15 min post OXY treatment, there is a delineation between conditions where the BOLD signal change for WT is significantly greater than MuKO and MuKO is significantly greater than vehicle.

### On-target effects of OXY on the pain circuitry

**Figure 4:**
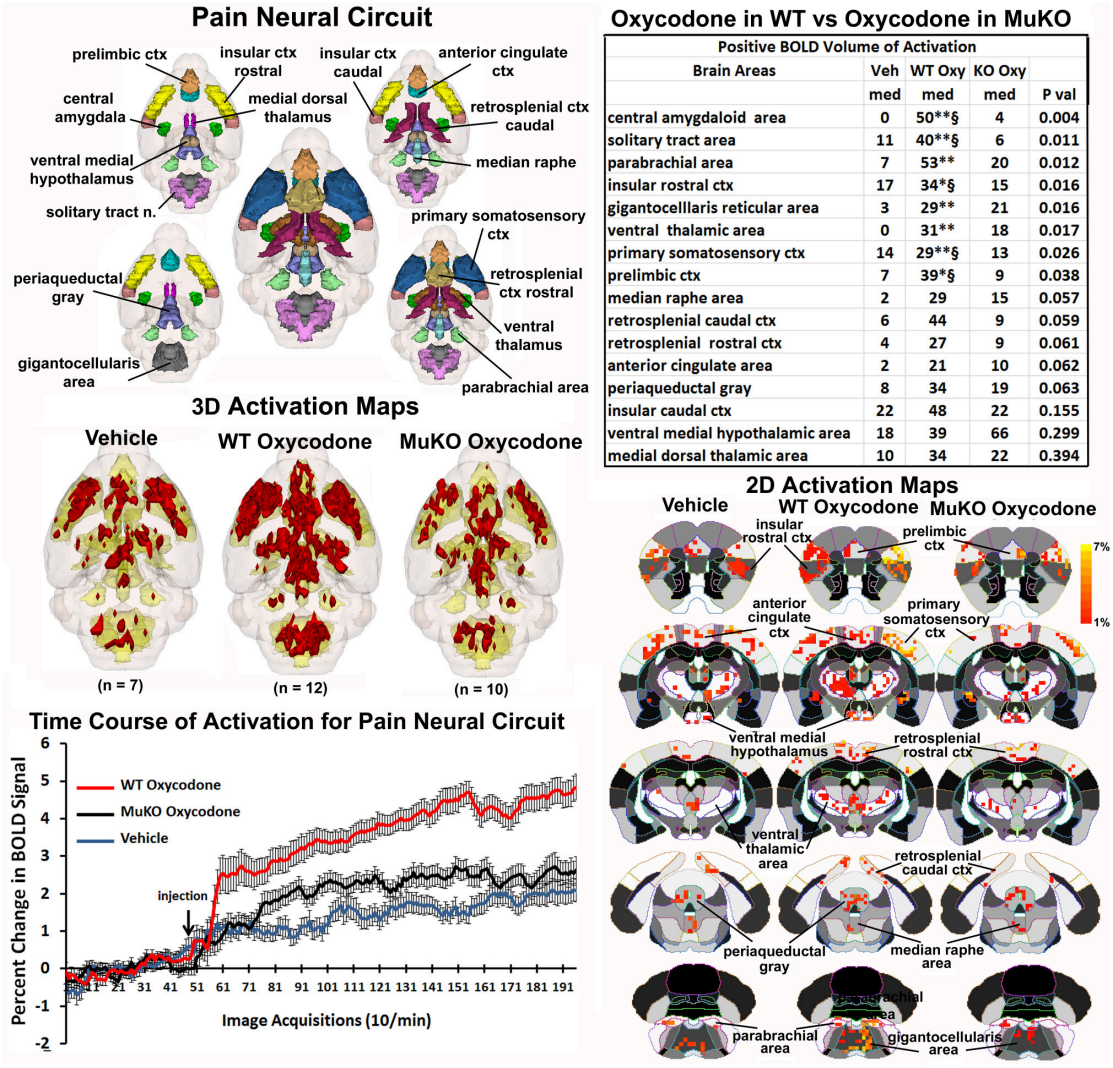
**Putative pain neural circuit**. Shown is a 3D color representation of the 16 different brain areas comprising the putative pain neural circuit. The layout and the description of the table and three composite figures are the same as Figure 3. The table of these 16 areas reports there were no significant differences in the volume of activation between vehicle (Veh) and MuKO (KO Oxy) conditions. WT mice given OXY showed significance from Veh (^*^p <0.05, ^**^p <0.01) and from MuKO conditions (§*p* <0.05).

The μ receptor has a major role in mediating the analgesic effects of opioids in mice as tested in the hot plate nociceptive assay (Baamonde et al., 1991; Kieffer and Gavériaux-Ruff, 2002). To assess the effect of OXY on changes in BOLD signal intensity related to analgesia, a putative pain neural circuit was reconstructed as shown in Figure 4. The pain neural circuit was adopted from Gauriau and Bernard using the parabrachial nucleus as key node (Gauriau and Bernard, 2002). The 16 volumes that comprise the pain neural circuits are color coded and annotated as reported in Figure 3 and coalesced into a single volume (yellow) below showing 3D activation maps for each experimental condition. These 3D data in 3D space can be viewed as 2D activation maps depicted in the lower right showing the precise location of the positive voxels registered to the MRI mouse atlas. WT mice treated with OXY showed a pattern of positive BOLD activation much greater than WT mice given saline vehicle or MuKO mice treated with OXY, while there appears to be little difference between vehicle and MuKO OXY conditions. Indeed, the table in the upper right reports that eight out of the sixteen brain areas comprising the pain neural circuit are significantly different. The volume of activation was significantly higher for WT OXY mice as compared to vehicle in all eight brain regions. However, unlike the activation pattern in the μ-opioid neural circuit, WT OXY mice showed significantly higher BOLD signal activation as compared to MuKO OXY mice in the areas of the central amygdala, solitary tract, insular, prelimibic and somatosensory cortices.

The change in BOLD signal over time for each of the three experimental conditions is shown in the lower left and clearly shows a delineation between each (group × time: *F*_(2, 398)_ = 5.40, *p* <0.0001). As in the time course data shown in Figure 1, all experimental groups showed a significant increase above baseline within 1 min of IP injection. WT OXY diverged and remained separate from MuKO OXY at repetition 61 (*p* = 0.038). WT OXY diverged and remained separate from vehicle at repetition 77 (*p* = 0.019). MuKO OXY started to diverge and remained relatively separate from vehicle at repetition 89 (*p* = 0.0002).

### Off-target effects of OXY

The data focusing on the μ-opioid neural circuit and the pain neural circuit in Figures 3, 4 show no significant differences between WT mice given saline vehicle and MuKO mice given OXY, demonstrating that most observed BOLD signal activation in WT mice reflect on-target (μ receptor receptor-mediated) OXY effects in live animals. However, from the total 122 brain areas there are six that show a significant difference in positive BOLD between the vehicle and MuKO OXY conditions (**see** Table 3Sp in Supplementary Material comparing Vehicle vs. MuKO OXY). The areas of difference are the facial nucleus of the medulla oblongata, 2nd and 4th cerebellar lobules, medial amygdala, medial preoptic area, and lateral geniculate. This small number of activated brain areas in MuKO mice treated with OXY compared to vehicle is in stark contrast to WT mice treated with OXY compared to vehicle (**see** Table 4Sp in Supplementary Material comparing Vehicle vs. WT OXY). In this latter case there are over 70 different brain areas showing activation.

**Figure 5:**
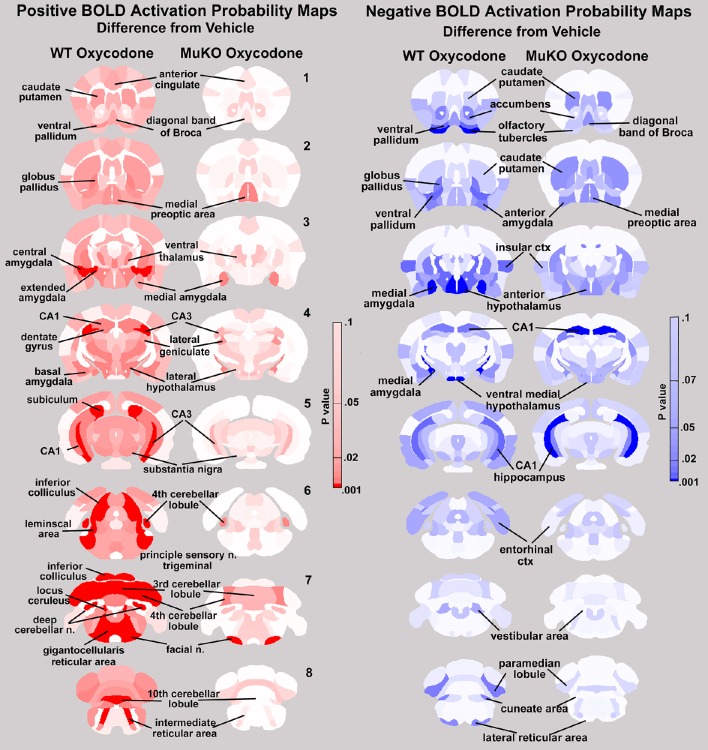
**Probability heat maps for positive and negative BOLD**. The probability maps generated as the significant difference between WT and MuKO mice given OXY as compared to WT mice given saline vehicle are present for positive BOLD (red) and negative BOLD (blue).

This relative absence of positive BOLD in MuKO mice given OXY is not so with negative BOLD. Both WT and MuKO mice given OXY showed many areas of the brain having a significant decrease in BOLD signal intensity as compared to vehicle (**see** Tables 3Sn, 4Sn). These differences between positive and negative BOLD in WT and MuKO mice given OXY as compared to vehicle are clearly seen in the probability heat maps shown in Figure 5. The red heat maps show many areas of high intensity for WT mice given OXY while only those few noted above for MuKO OXY, (full list pf ROI in Supplementary table 5Sp). The blue heat maps for negative BOLD show many areas of high intensity for both experimental conditions. Interestingly, there are clear differences. For example MuKO mice given OXY show a pronounced decrease in negative BOLD signal in the caudate/putamen and CA1 of the hippocampus as compared to WT OXY. Conversely, WT mice given OXY show a pronounced decrease in negative BOLD signal in the olfactory tubercles, ventral medial hypothalamus, medial amygdala, ventral pallidum, paramedian lobule of the cerebellum, and lateral reticular area of the brain stem (see Table 5Sn in Supplementary Material for complete ROI list).

## Discussion

The three opioid receptors are located throughout the brain across all mammalian species. Opioid signaling in the brain involves the regulation of pain, and the modulation of behavior associated with reward, addiction, depression and anxiety [for reviews, see Al-Hasani and Bruchas, 2011; Pradhan et al., 2012; Lutz and Kieffer, 2013a,b]. Any change in brain activity in response to the systemic, IP administration of OXY as reported here, could be due to the direct action of drug on any of these brain opioid receptors, with preferred action at the μ receptors, or via yet unknown off-target effects or indirect via other neurotransmitter systems such as dopamine (Vander Weele et al., 2014) and GABA (Takasu et al., 2015). Comparing the pattern of BOLD signal change in WT and MuKO mice in response to OXY provided us with an opportunity to evaluate the significance of the μ opioid receptor in mediating OXY's effects on the brain in the awake mouse.

In brief, the pattern of positive BOLD showed activation across many brain areas (72 out of 122) in WT mice within 15 min of OXY administration. The OXY-induced positive BOLD was essentially eliminated in MuKO mice, demonstrating a majority of on-target responses. A few exceptions were cerebellum (lobules 2, 3, 4, and 10 and deep cerebellar nuclei), CA3 of the hippocampus and medial amygdala and preoptic areas, where receptor expression is low or absent in WT mice, revealing off-target effect at some brain sites. OXY also caused an increase in negative BOLD in WT mice, however this was observed in only few areas (16 out of 122). These areas included the basal ganglia (accumbens, olfactory tubercles, ventral pallidum, and globus pallidus) insular ctx, anterior and ventral medial hypothalamus, anterior and medial amygdala, entorhinal ctx, CA1 of the hippocampus and the paramedian lobule of the cerebellum. Unlike the positive BOLD response to OXY in the MuKO mice the negative BOLD was only partially eliminated and in some case intensified. Areas that were reduced included the basal ganglia, hypothalamus, amygdala, and cerebellum. Areas that were intensified include the caudate/putamen, diagonal band of Broca, medial preoptic area and CA1. Overall, the data show that most of OXY-induced changes in positive and negative BOLD are mediated through the μ receptor. Further, activation and deactivation events detected in the absence of the μ receptor in a few areas, also reveal the existence of off-target OXY effects that may be mediated by κ and δ opioid receptors, or other non-opioid unknown target (Yoburn et al., 1995). Looking at Tables 1, 2 for the significant changes in positive and negative BOLD signal intensity, respectively, it is obvious that OXY, acting through the μ receptor, has a global effect on brain activity that reaches beyond those areas with a high density of receptor highlighted in Figure 3. Indeed, autoradiographic receptor binding on this mouse line shows a widespread distribution of μ receptor (Kitchen et al., 1997).

The pattern of BOLD activation observed in these studies with OXY is similar to the opioidinduced changes reported in human functional imaging studies using positron emission tomography (PET) and fMRI (Adler et al., 1997; Petrovic et al., 2002; Wise et al., 2002; Wagner et al., 2007; Becerra et al., 2013; Seah et al., 2014). For example, the activity of remifentanil, a short acting specific μ receptor agonist was tested in humans with BOLD imaging, and as in this study, the focus was on brain areas known to have a high density of opioid receptors and brain areas involved in pain processing (Leppä et al., 2006). As expected limbic cortex, insula, and anterior cingulate and their thalamic inputs together with amygdala and hippocampus were activated with remifentanil exposure. The cerebellum was also activated. To date, the only human imaging study testing OXY examined resting-state functional connectivity following oral administration of drug (Gorka et al., 2014). Functional correlates were examined between seed areas in the anterior cingulate and brain areas associated with pain and showed OXY reduced coupling between cingulate and insula/putamen.

While there have been several published reports using fMRI to follow opioid-induced changes in brain activity in rodents (Xi et al., 2004; Shah et al., 2005; Liu et al., 2007), all have been conducted under anesthesia which significantly limits both the degree of signal change and pattern of activation (Borsook et al., 2006; Ferris et al., 2011; Liang et al., 2011; Seah et al., 2014; Haensel et al., 2015). A previous fMRI study that most closely parallels the findings reported here was that done in awake rats in response to buprenorphine (Becerra et al., 2013). Awake rats showed activation in the limbic and olfactory cortices, caudate-putamen, globus pallidus, septum, hippocampus, thalamus, PAG, inferior colliculus, and cerebellum. The same areas were activated in the awake mouse in response to OXY as reported here. Also, buprenorphine caused a decrease in BOLD signal in the awake rat. The negative BOLD was found in the thalamus, hypothalamus, and hippocampus.

OXY abuse is well documented, much of which can be attributed to drug dependence and withdrawal as reported in clinical (Jones et al., 2011; Mars et al., 2014) and preclinical studies (Hutchinson et al., 2009; Wiebelhaus et al., 2016). In addition, OXY has reinforcing effects as measured by self-administration and condition place preference studies in rodents (Beardsley et al., 2004; Zhang Y. et al., 2009; Rutten et al., 2011; Bryant et al., 2014; Zhang et al., 2015). The mesocorticolimbic dopaminergic system is a key pathway involved in drug reinforcement (Koob, 1992) and as such, we anticipated OXY would affect activity in this neural circuit. While there was a significant increase in positive BOLD in the ventral tegmental area, prelimbic ctx, and ventral pallidum we were surprised to see no activation in the shell of the accumbens and a significant negative BOLD response in the core of the accumbens. However, these data are consistent with the hypothesis that the rewarding effect of drugs are coded by the activity in the medium spiny GABAergic neurons in the accumbens (Carlezon and Thomas, 2009). Medium spiny neurons make up over 90% of the neurons in the accumbens (Meredith, 1999). Electrical recordings from these neurons show opioids inhibit their activity (McCarthy et al., 1977; Hakan and Henriksen, 1987). This suppression of activity over a majority of the neurons in the accumbens might appear as no change in BOLD signal intensity or a negative BOLD. Indeed, humans given remifentanil show no activation of the accumbens with BOLD imaging (Leppä et al., 2006). The same is true in BOLD imaging studies using buprenorphine (Becerra et al., 2013).

In a series of studies, Shih and collaborators characterized a counter-intuitive negative BOLD signal change in the striatum of the rat in the face of increased neuronal activity caused by pain and exacerbated by morphine treatment during a noxious stimulus (Shih et al., 2009, 2011, 2012). The effect of morphine was blocked with naloxone pretreatment, evidence of opioid receptor involvement. This phenomena is associated with a dopamine-induced vasoconstriction that can be blocked by the D2/D3 receptor antagonist eticlopride or disruption to the dopaminergic inputs from the substantia nigra (Chen et al., 2005; Shih et al., 2009). The pronounced negative BOLD in WT OXY mice in areas of the basal ganglia (e.g., ventral pallidum, globus pallidus, olfactory tubercles, and to a lesser extent the caudate putamen) would support this dopamine-mediated response observed in this awake mouse imaging study (**see** Figure 3).

Interestingly, the caudate/putamen still showed a heightened negative BOLD in response to OXY in the MuKO mice suggesting a mechanism independent of the μ-opioid receptor. Whether this is mediated through the k or δ opioid receptors or involves yet another target is unknown.

Opioid agonists act by decreasing calcium conductance and increasing potassium conductance with the net effect of decreasing membrane excitability (Di Chiara and North, 1992) predominantly in GABAergic neurons (Vaughan et al., 1997). The classically described disinhibition effect of opioids results in the activation of dopaminergic transmission within reward pathways, as well as activation of endogenous descending antinociceptive systems involving key opioid sensitive nodes in the PAG, median raphe and rostral ventromedial medulla (RVM). The mouse 3D MRI atlas used in these studies shows the RVM as the gigantocellularis and paragigantocellaris areas bounded by the facial nucleus in the medulla (Figure 3) (Lau and Vaughan, 2014). This general area of the medulla showed a pronounced increase in positive BOLD signal intensity following OXY in WT but not MuKO mice corroborating the importance of the μ receptor in opioid analgesia. The antinociceptive action of the opioid agonist morphine in mice is exclusively mediated through the μ receptor (Matthes et al., 1996; Sora et al., 1997). Indeed, many of the brain areas shown in the putative pain neural circuit (Figure 2) were activated by OXY in WT but showed little activity in MuKO mice. However, in a study by Ross and Smith (Ross and Smith, 1997) it was reported the direct administration of OXY into the cerebroventricular system of rats has intrinsic antinociceptive effects that cannot be blocked by prior treatment with selective μ or δ receptor antagonists. The authors concluded that OXY's analgesic activity is mediated through the κ receptor. There are no data from this study that would contest that finding. The present studies were designed to finger print the OXY-induced pattern of brain activity and the contribution of the μ receptor in that response. Indeed it would be interesting to use BOLD imaging to study the response to a painful stimulus during vehicle and OXY challenge. While much of the OXY-induced BOLD activity is absent in the MuKO mouse, this may reveal endogenous μ opioid receptor-mediated mechanisms underlying pain processing and the reduction of pain perception.

It has been known for some time that the cerebellum, in addition to its role in motor coordination, is also involved in cognitive and emotional experience (Haines et al., 1984; Turner et al., 2007; Perciavalle et al., 2013). This acceptance of cerebellum into the neural circuitry of affective behavior has recently extended to include addiction (Miquel et al., 2016). This is made possible by the many reciprocal connections between the cerebellum, hypothalamus, limbic cortex, amygdala, and hippocampus (Snider and Maiti, 1976; Heath et al., 1978; Haines et al., 1984; Rogers et al., 2011) and the presence of opioid receptors in the cerebellum. The human cerebellum has both μ and κ opioid receptors but not δ (Schadrack et al., 1999). The many studies on opioid receptor localization in the CNS of rodents (Atweh and Kuhar, 1977; Goodman et al., 1980; Moskowitz and Goodman, 1984; Tempel and Zukin, 1987; Sharif and Hughes, 1989; Mansour et al., 1994a,b; 1995) are all in agreement as to the absence of μ and δ receptor in the cerebellum. While many fail to report the presence of κ receptor, Temple and Zukin showed autoradiographic evidence of κ binding in the cerebellum of the rat (Tempel and Zukin, 1987), a finding corroborated by Sharif and Hughes in rat and guinea pig (Sharif and Hughes, 1989). In light of these data, the OXY-induced activation of the cerebellum in MuKO mice would suggest a possible interaction with the κ receptor, or again a non-opioid off-target effect of OXY. In the future, the analysis of OXY effect in κ opioid receptor KO mice may be of interest to identify whether κ receptormediated mechanisms contribute to the OXY BOLD activation map.

### Caveats and data interpretation

For any imaging study on awake animals the issues and consequences related to the stress of head restraint and restricted body movement must be considered. Protocols have been developed to help lessen the stress of an imaging study by acclimating animals to the environment of the MR scanner and the restraining devices helping to reduce stress hormones levels and measures of sympathetic autonomic activity (Zhang et al., 2000; King et al., 2005). These acclimation procedures put animals through several simulated imaging sessions and have been used to study sexual arousal in monkeys (Ferris et al., 2004), generalized seizures in rats and monkeys (Tenney et al., 2003, 2004), and exposure to psychostimulants like cocaine (Febo et al., 2004, 2005; Ferris et al., 2005), amphetamine (Madularu et al., 2015), nicotine (Skoubis et al., 2006) and apomorphine (Zhang et al., 2000; Chin et al., 2006). Nonetheless, one must consider the experimental confound that exists with low levels of arousal and stress associated with imaging awake animals.

Another consideration when interpreting the data is the morphological differences in brain structure that may occur between WT and transgenic rodents. This raises the possibility that regional differences in brain volume may have influenced the BOLD signal analysis particularly when the data is reported as volume of activation i.e., number of voxels activated in a 3D brain volume. While there was little difference in whole brain volume and the volumes of individual brain areas in between these WT and MuKO mice we controlled for this possibility by normalized the volume of activation to the brain volume of interest for each subject prior to statistical comparisons for both genotypes.

As noted above, the opioid receptors μ, κ, and δ are located throughout the brain, but they are also found in the periphery localized primarily to the enteric nervous system of the gut (Holzer, 2009) and less so in the heart (Sobanski et al., 2014). Opioids have a dramatic influence on gut motility with the unwanted side effect of constipation (Holzer, 2009) and can attenuate the sympathetic regulation of cardiac function (Wong and Shan, 2001). Viscero-sensory information from these organs would be conveyed back to the brainstem through spinal cord and cranial nerves to affect BOLD signal intensity in many areas in the hindbrain associated with autonomic function. The nucleus of the solitary tract (NTS), parabrachial and gigantocellularis nuclei and reticular formation, key areas in the integration of viscero-sensory information, have direct sensory connections with spinal neurons (Menétrey and De Pommery, 1991). This raises the question, how much of the activity in the brain is direct or mediated through the periphery? Indeed any change in brain activity in response to the systemic, IP presentation of OXY as reported here, could be due to the direct action of drug on the brain opioid receptors or indirectly through peripheral nervous system.

In conclusion, our approach permits establishing opioid-induced BOLD activation maps in awake mice. In addition, comparison of WT and MuKO mutant mice reveals both on-target and off target activation events, and set an OXY brain signature that should, in the future, be compared to other μ opioid agonists.

## Author contributions

KM: performed the study (ran scans), wrote manuscript. DM: analyzed data, wrote manuscript. SI: ran scans. JY: analyzed data, wrote manuscript. PK: analyzed data, developed analysis pipeline. ED: generated mouse lines. BK: wrote manuscript, devised experiment. CF: wrote manuscript, analyzed data, devised experiment.

## Funding

We thank the US National Institutes of Health (National Institute of Drug Addiction, grant #05010 and National Institute on Alcohol Abuse and Alcoholism, grant #16658.

### Conflict of interest statement

CF has a financial interest in Animal Imaging Research, the company that developed the mouse imaging system. CF and PK have a financial interest in Ekam Solutions the company that developed the mouse MRI atlas. The other authors declare that the research was conducted in the absence of any commercial or financial relationships that could be construed as a potential conflict of interest.

## Supplementary material

Table 1S **Positive and negative BOLD activation for 122 brain areas**. Shown is a list of 122 brain areas and their median (med) number of voxels as a percentage of the total Brain Area volume (i.e., number of voxels activated, divided by the total number of voxels in the 3D volume of interest, times 100) for wild-type given saline vehicle (Veh) wild-type given oxycodone (WT Oxy) and μ receptor knock-out mice given oxycodone (KO Oxy). These volumes of activation for each brain region across genotypes were analyzed using a Newman-Keuls multiple comparisons test statistic and ranked in order of their significance. *P*-values are presented on the far right column.

Table 2S **Negative BOLD activation for 122 brain areas**. Same as Table 1S.

Table 3Sp **Positive BOLD volume of activation veh vs. MuKO oxycodone**. Shown is a list of 122 brain areas and their median (med), maximum (max), and minimum (min) number of voxels as a percentage of the total Brain Area volume (i.e., number of voxels activated, divided by the total number of voxels in the 3D volume of interest, times 100) for wild-type given saline vehicle (Vehicle) and μ receptor knock-out mice given oxycodone (MuKO Oxy). These volumes of activation for each brain region between groups were analyzed using a Wilcoxon rank-sum test.

Table 3Sn **Negative BOLD volume of activation veh vs. MuKO oxycodone**. Same as Table 3Sp but negative BOLD volume of activation.

Table 4Sp **Positive BOLD volume of activation veh vs. WT Oxycodone**. Same as Table 3Sp.

Table 4Sn **Negative BOLD volume of activation veh vs. WT Oxycodone**. Same as Table 3Sp but negative BOLD volume of activation.

Table 5Sp **Positive BOLD volume of activation WT oxycodone vs. MuKO oxycodone**. Same as Table 3Sp.

Table 5Sn **Negative BOLD volume of activation WT oxycodone vs. MuKO oxycodone**. Same as Table 3Sp but negative BOLD volume of activation.

Figure 1S **Volumetric analysis**. Shown are bar graphs for the total brain volume. Vertical lines denote SEM.
