# Supplementary figures and images for: BOLD Imaging in Awake Wild-Type and Mu-Opioid Receptor Knock-Out Mice Reveals On-Target Activation Maps in Response to Oxycodone
Source: Front Neurosci. 2016 Nov 3;10:471. doi: 10.3389/fnins.2016.00471 (PMC5094148; doi:10.3389/fnins.2016.00471)

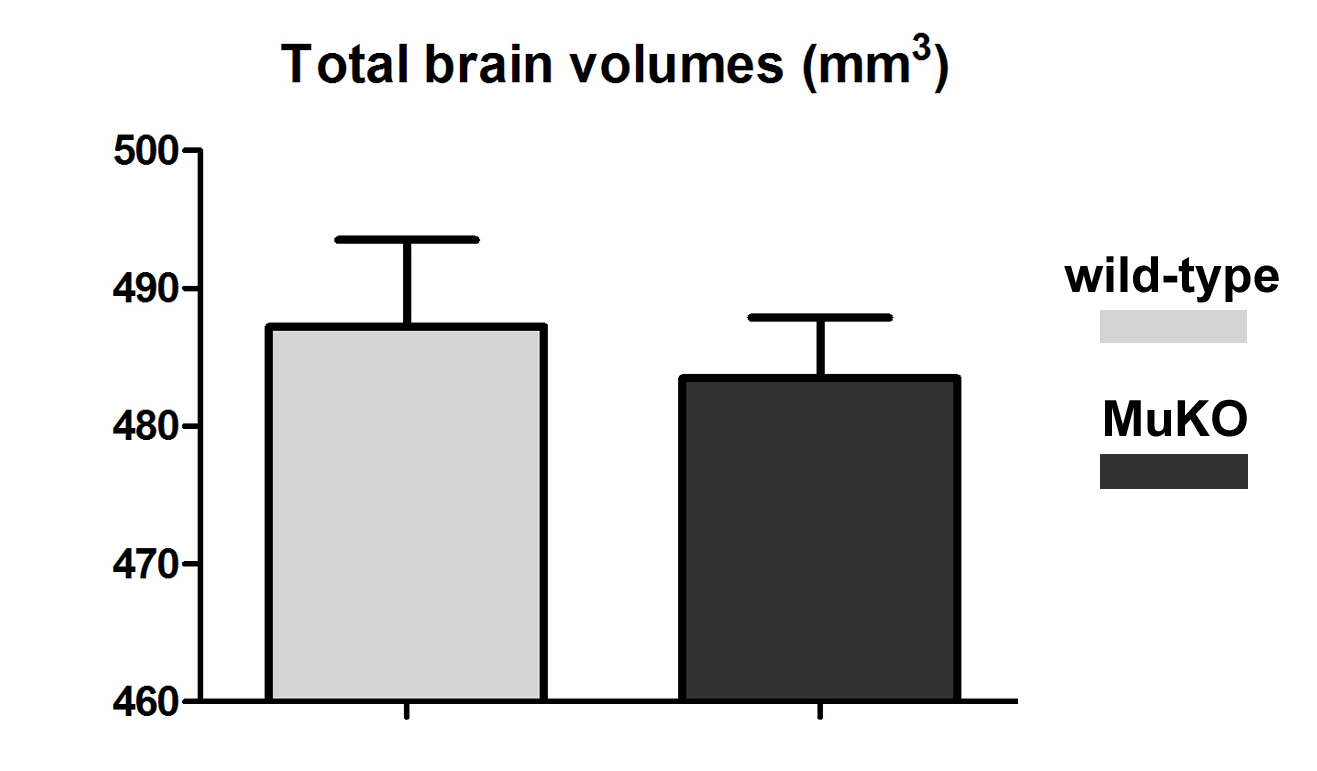

Supplement: Figure 1S — Volumetric analysis. Shown are bar graphs for the total brain volume. Vertical lines denote SEM. [file Image1.TIF]
